# What is ancestry?

**DOI:** 10.1371/journal.pgen.1008624

**Published:** 2020-03-09

**Authors:** Iain Mathieson, Aylwyn Scally

**Affiliations:** 1 Department of Genetics, Perelman School of Medicine, University of Pennsylvania, Philadelphia, Pennsylvania, United States of America; 2 Department of Genetics, University of Cambridge, Cambridge, United Kingdom; University of California, Los Angeles, UNITED STATES

Ancestry connects genetics and society in fundamental ways. For many people it has cultural, religious or even political significance, and can play a key role in shaping personal and public identities. People’s desire to discover their own ancestry drives the multibillion-dollar genealogy industry, which has grown rapidly in the era of consumer genomics. Companies such as 23andMe and Ancestry now claim tens of millions of customers worldwide. In parallel, our scientific understanding of the human past is being transformed by studies of ancient and modern genetic data, which allow us to track changes in ancestry over space and time. Sophisticated methods have been developed to infer and visualise these relationships. Thus, it seems that both scientists and the wider public are learning more and more about ancestry, and there is an optimistic sense that genetic data provide an exhaustive repository of ancestral information.

However, although frequently discussed, ancestry itself is rarely defined. We argue that this reflects widespread underlying confusion about what it means in different contexts and what genetic data can really tell us. This leads to miscommunication between researchers in different fields, and leaves customers open to spurious claims about consumer genomics products and overinterpretation of individual results.

In wider usage, the terms ancestry and ancestors often indicate a general connection to people or things in the past. But in a genetic context they have a more specific meaning: your ancestors are the individuals from whom you are biologically descended and ancestry is information about them and their genetic relationship to you. Even here however, confusion arises from the way that ancestry is presented and discussed. Rather than emphasising its complex structure, results are often simplified in terms of discrete categories. While convenient and sometimes useful, ultimately this is misleading about the nature of ancestry. These labels can also impose contemporary political or cultural divisions which may be misrepresentative of ancestral relationships.

Another source of confusion is that three distinct concepts–genealogical ancestry, genetic ancestry, and genetic similarity–are frequently conflated. We discuss them in turn, but note that only the first two are explicitly forms of ancestry, and that genetic data are surprisingly uninformative about either of them. Consequently, most statements about ancestry are really statements about genetic similarity, which has a complex relationship with ancestry, and can only be related to it by making assumptions about human demography whose validity is uncertain and difficult to test.

*Genealogical ancestry* probably reflects the most common and intuitive understanding of the term ancestry. Consider your parents, grandparents, or even great-grandparents. You likely have a sense of these people as individuals, even if you have never met them. If one of them belonged to a particular group X, you might say that you have some “X” ancestry. You might even be able to claim ancestry in this way from more distant ancestors, based on historical or genealogical records. Thus genealogical ancestry is defined in terms of identifiable ancestors in your family tree or *pedigree*. Often it may be quantified; for example, if one of your eight great-grandparents belonged to X you might describe yourself as “one eighth X”. N generations ago you have at most 2^N^ genealogical ancestors, and if some proportion of them belonged to X you might claim that proportion of ancestry from X.

**Fig 1:**
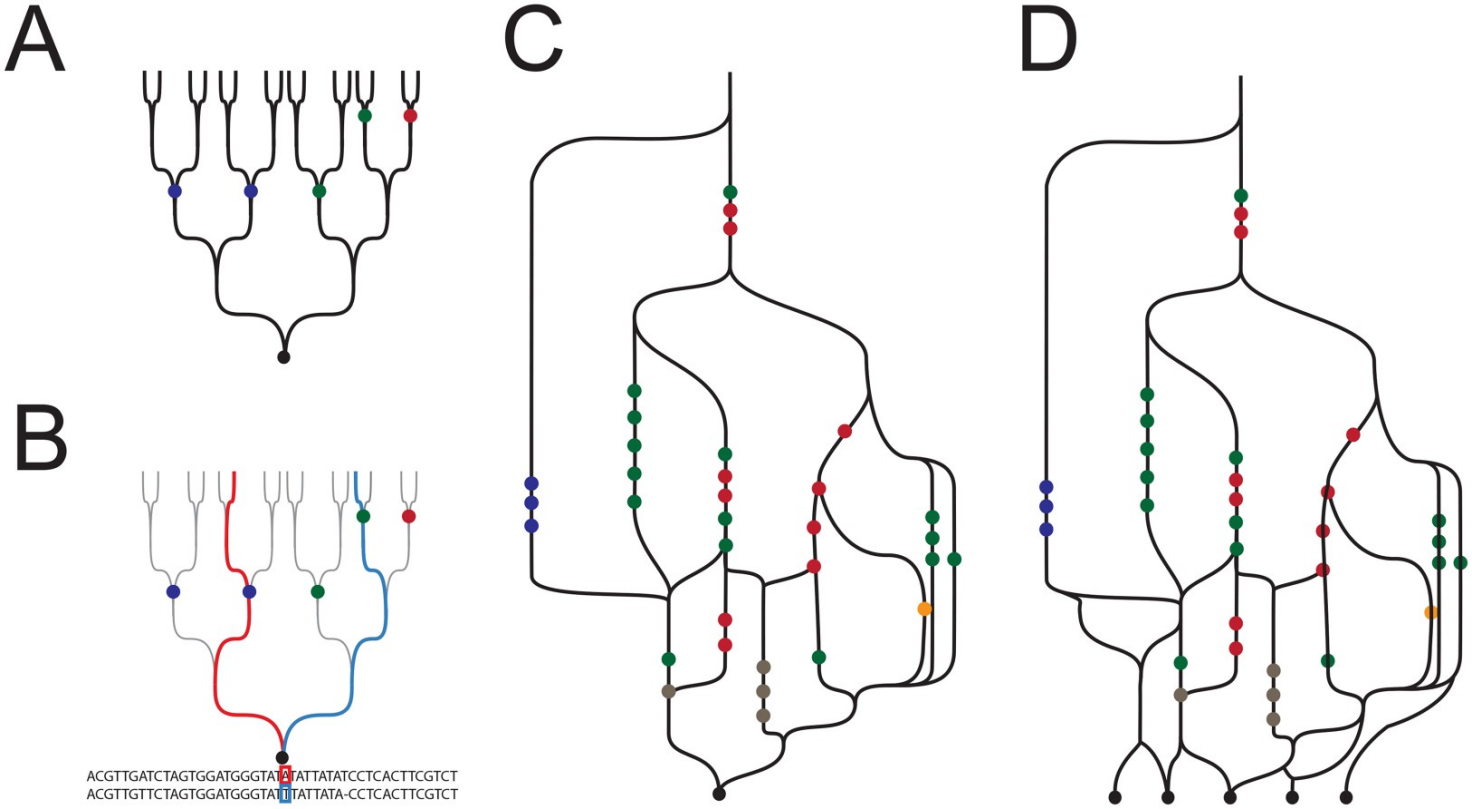
Genealogical and genetic ancestry. **A**: The pedigree of a single individual. Circles indicate specific ancestors that could be used to define ancestry categories. **B**: At any single position in the diploid genome, genetic ancestry over the past N generations traces two paths (red and blue) through the, at most, *2*^*N*^ available. **C**: Genetic ancestry in the form of the ARG for a single individual. Combining genetic ancestry from different positions leads to a graph, incorporating all realized genetic ancestry paths, implicitly passing through points representing specific individuals. The ARG is contained within the structure of the pedigree, with nodes corresponding to ancestors in which there was a recombination or coalescence event, and edges or lines between them representing paths of descent (through other ancestors which are not represented) for particular segments of DNA. **D**: The ARG is usually used in the context of the merged ARGs of multiple individuals.

There are two concepts here: the pedigree, which specifies how all your genealogical ancestors are related to each other, and the ancestry category ‘X’ which ascribes a characteristic of interest to some of them. The pedigree can be thought of as a graph, with nodes representing your genealogical ancestors connected by edges representing parent-child relationships between them (Fig 1A). Were we able to draw it in full it would be impracticably large, but in principle from a pedigree alone we can deduce facts about relatedness, for example that Charles Darwin’s wife Emma was his first cousin (and therefore approximately one eighth of their genomes were identical). Importantly, whereas the pedigree is fixed, ancestry categories can be arbitrary, reflecting aspects of ancestry that we happen to be interested in. X for example could be “British”, “English”, “Huguenot”, or any label referring to culture, geography or some other aspect of an individual’s identity.

Thus, to describe your genealogical ancestry requires knowledge of your ancestors, the pedigree relating them to you, and sufficient information to assign them to categories of interest. In practice however, few people have comprehensive knowledge of their ancestors beyond a handful of generations ago. Even when researching their genealogy, people tend to focus on a small number of lineages for which records exist or which are of particular interest, neglecting the exponential growth in the number of genealogical ancestors back in time. Genetic data can help with this limitation through *genetic genealogy*, which identifies relatives based on the distinctive patterns of genetic variation they share. Knowledge of your relatives, while not ancestry in itself, can facilitate the pooling of information about shared ancestors in combination with traditional genealogical information. However, this information may still be difficult to obtain. This limitation raises the question of whether there is a form of ancestry that could be learned from genetic data alone.

The natural definition of this kind of ancestry is *genetic ancestry*, which differs from genealogical ancestry in that it refers not to your pedigree but to the subset of paths through it by which the material in your genome has been inherited. Because parents transmit only half their DNA to offspring each generation, an individual’s genetic ancestry involves only a small proportion of all their genealogical ancestors [1,2]. At any given position in one of your chromosomes, your DNA is inherited through one of the many possible paths through your pedigree (Fig 1B). Different positions in the genome may have different paths of inheritance, because parental chromosomes are shuffled together during meiotic recombination. Thus the difference between genealogical and genetic ancestry can be summed up by the observation that full siblings have identical genealogical ancestry but differ in their genetic ancestry, due to differences in the transmission of chromosomal segments from their parents.

The fundamental representation of genetic ancestry is a structure called an *ancestral recombination graph* (ARG; Fig 1C) [3]. The ARG is central to population genetics, and many methods for making inferences about demographic history proceed by either implicitly or explicitly reconstructing ARGs [4–7]. Recalling the graph structure of an individual’s pedigree, the ARG is a subset of the pedigree representing the ancestry of the DNA inherited by that individual. It contains only those edges along which inherited segments of DNA have been transmitted, and only those nodes corresponding to ancestors in which there was a recombination or coalescence event. The ARG therefore tells you which parts of your genome were inherited from which ancestors, and represents all the ancestral information that can be obtained from genetic data alone. For example, your pedigree includes many ancestors from whom you inherited no genetic material, but such ancestors are not included in the ARG, and your genome cannot provide any information about them. The ARG can also be used to represent the genetic ancestry of a sample of multiple individuals by merging the individual ARGs into a single graph (Fig 1D).

Just as for genealogical ancestry, we may want to summarize the genetic ancestry of an individual in terms of particular groups or categories of interest. If we could identify specific ancestors in the ARG (Fig 1C) then, analogous to genealogical ancestry, we could say that an individual has genetic ancestry in a given category if any edge in his or her ARG passes through an ancestor in that category. In other words, genetic ancestry in category X means that some fraction of an individual’s genome is inherited directly from an ancestor in X. Genetic ancestry in X implies genealogical ancestry in X, but not vice versa. And as with genealogical ancestry, we could extend this approach to summarizing genetic ancestry by counting the proportion of an individual’s genome inherited from ancestors in X.

One factor motivating interest in particular ancestors or categories may be the idea that such ancestry is associated with genetic effects on certain traits. Whether this is plausible or not, genetic ancestry appears to provide an essentialist notion of ancestry that excludes any relationship that does not correspond to an inherited DNA segment. However, it turns out that determining genetic ancestry is even less practical an idea than genealogical ancestry. Whereas at least some of the pedigree may be pieced together from genealogical records, the ARG must be inferred solely from patterns of genetic variation–a very challenging problem (despite impressive recent progress [7,8]). Even if we could reconstruct the true ARG and the ancestors on each edge, we would have the same problem of needing information about membership in specific ancestry categories in order to give meaningful summaries.

The impracticality of fully determining either form of ancestry means that most analyses take an alternative approach. Typically, they aim to infer an approximate summary of genetic ancestry without reconstructing the ARG. For example, researchers may be interested in the demographic relationships between human populations but not necessarily the details of individual ancestors. Perhaps the closest we can practically get to this is an *admixture graph* [9,10], which relies on a concept of “population ancestry”, embedded in a graph which is similar to the ARG but relates populations rather than individuals. In any real population, individuals will differ in their ancestry and the true ARG will be extremely complex. The admixture graph focuses on the idea that, when averaged over the whole genome, these differences can be approximated as varying proportions of ancestry from multiple source populations. Since populations are explicitly represented as nodes in the admixture graph, it is more straightforward to attribute ancestry categories to them (compared to an ARG or pedigree), which makes this an appealing way to summarize ancestry. However there are drawbacks: populations may be poorly sampled or include unrepresentative individuals, or they may not correspond to identifiable groups. It is hard to know whether the inferred sources of ancestry (sometime called “ghost populations”) are real but unsampled populations or simply algorithmic constructs representing a simplification of more complex demography. More fundamentally, admixture graphs enforce the idea of discrete populations in a way which is at odds with the complexity of human demographic structure. For now they remain rare outside the population genetic literature, and care is needed in presenting them to a wider audience, as they represent an abstraction of ancestral demography which can easily be misinterpreted as something more concrete.

**Fig 2:**
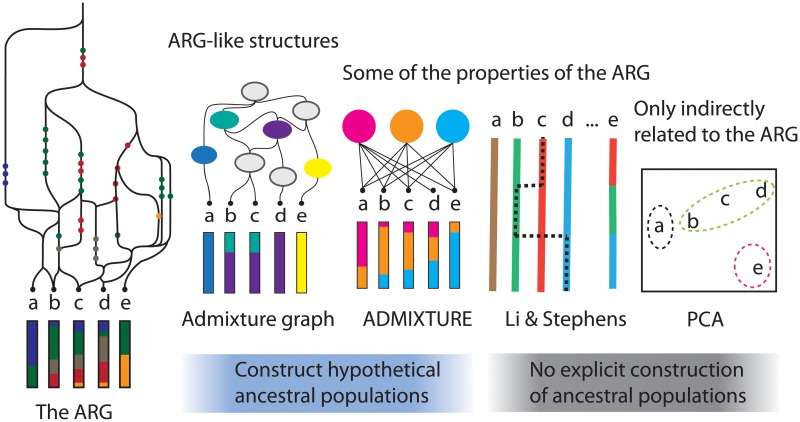
Statistical summaries of genetic data. The most popular approaches range from approximate representations of the ARG (e.g. admixture graphs), to summaries of data with no direct relationship to genetic ancestry (e.g. PCA).

More commonly, when geneticists and consumer genomics companies talk about ancestry they are really talking about *genetic similarity* between populations and individuals. For example, the output of methods that summarize genetic variation among samples such as principal component analysis (PCA), ADMIXTURE [11] (an implementation of the STRUCTURE model [12]) and Chromopainter [13] (based on the Li & Stephens haplotype copying model [14]) are frequently interpreted in terms of ancestry. Some of these methods allow individual genomes to be represented as combinations of reference populations, which are either explicitly defined in terms of other individuals in the dataset, or constructed implicitly as part of the algorithm. These are ‘ancestry-like’ relationships, and since the ARG contains all the information about the evolutionary genetic process which produced the differences between samples, the outputs of these methods can be seen as summaries of the ARG (Fig 2).

Most customers of ancestry testing companies are not concerned about technical distinctions between genealogical ancestry, genetic ancestry and genetic similarity. And certainly, genetic similarity can be informative about genetic ancestry on a broad temporal and geographical scales. For example, if segments of your genome are found to be similar to individuals from particular continental groups (“European”, “African”, “Native American”), it is very likely that you have genetic ancestry from those groups in the past few hundreds or thousands of years. However this masks important caveats. Implicitly here we are making use of non-genetic information, for example historical information about population and group continuity in these regions. The geographical connection between ancestry and genetic similarity becomes less clear as we move to more recent or finer-scale structure. For example, at sub-continental scales we are often much less confident about the continuity of populations over the past few thousand years. Emerging evidence from ancient DNA emphasizes discontinuity, with many populations experiencing multiple episodes of replacement over the past few thousand years [15]. As a result, some of your genetic similarity to present-day individuals in a particular country may derive from shared ancestry tens or hundreds of generations ago in a different part of the world. Describing fine-scale population structure in terms of ancestry (“your ancestors lived in Ireland”), rather than relatedness (“your relatives live in Ireland”) underestimates the contribution of migration to human demography.

A more practical issue is that measures of similarity are always sensitive to the choice and labelling of reference populations. Ancestry inferred from genetic similarity will emphasize and reify existing groups in ways which may hide important connections between populations and individuals, while giving the false impression that inference from genetic data is unbiased. A particularly persistent source of misinterpretation is the naming of ancestry categories based on their similarity to present-day populations. Examples include ADMIXTURE clusters named after the present-day population in which cluster membership is maximized, or internal admixture graph nodes named after the closest leaf node. Population geneticists may find it convenient to talk about such clusters as ‘real’ populations, as a shorthand and an aid to discussion of results. But this risks confusing those unfamiliar with the algorithmic nature of these clusters—which often means researchers in different fields. A related issue is that the usefulness of genetic similarity is limited by restricted or biased sampling of reference panels. This is particularly relevant for populations of non-European ancestry, which are under-represented in public and commercial databases. Individuals from such populations may find their ancestry attributed to related groups or regions that happen to be better represented, for example those with substantial representation in Europe or the USA. Thus recent social, economic and historical factors can further distort the picture of ancestry that arises from genetic similarity.

Ultimately, all descriptions of ancestry, whether approximate or based on reconstructing an ARG or complete pedigree, imply some arbitrary choices. Any summary in terms of an explicit or implicit set of categories involves a loss of information, because focusing on category X means ignoring the details of all ancestors not in X, as well as any ‘non-X’ aspects of the ancestors in X. Even a large set of categories spanning many past or present human cultures implicitly specifies a time period within which they are meaningful, and thus discards information at all other times. This reduction may be useful if it helps us focus on questions of interest. For example, we might be interested in the distribution of Neanderthal ancestry, in which case “Neanderthal” is a natural category to use and is obviously important for describing population structure 100,000 years ago, even though it does not emerge clearly from the genetic structure of present-day humans. Conversely, categories derived from present-day data may be misleading about or even irrelevant to ancient structure. There are unavoidable consequences to the categories we use, whether hand-picked or obtained from unsupervised clustering. Thinking about ancestry in terms of features we know biases us away from those we don’t, and which by that fact may be of most interest.

Finally, we emphasize that the role of genetic data in shaping cultural or ethnic identities is entirely a matter for the individuals and groups concerned. For example, genetic data might indicate the existence of an ancestor in a particular category, or confirm the identity of a particular ancestor, but this need not imply anything about membership in any group today. Equally, some groups and individuals may choose to regard genetic similarity to a reference population as a relevant aspect of their identity. But again, it is important to be explicit about what this represents, rather than treating it as a complete picture of genealogical or genetic ancestry. In both academic research and personal genomics, we should be clear about what we are measuring, the assumptions we make, and the surprisingly narrow limits of what genetic data can tell us about ancestry.
